# Countermovement Jump Force‐Time Mechanics Differentiate ACL Injury Status in Elite Alpine Ski Racers

**DOI:** 10.1111/sms.70270

**Published:** 2026-03-26

**Authors:** Nathaniel Morris, Ricardo da Silva Torres, Mark Heard, Patricia Doyle Baker, Walter Herzog, Matthew J. Jordan

**Affiliations:** ^1^ Integrative Neuromuscular Sport Performance Laboratory, Faculty of Kinesiology University of Calgary Calgary Alberta Canada; ^2^ Human Performance Laboratory, Faculty of Kinesiology University of Calgary Calgary Alberta Canada; ^3^ Wageningen Data Competence Center Wageningen University & Research Wageningen the Netherlands; ^4^ Banff Sports Medicine Centre Canmore Alberta Canada; ^5^ Sport Medicine Centre University of Calgary Calgary Alberta Canada

**Keywords:** classification algorithms, knee injury, power, rehabilitation, ski racing

## Abstract

Biomechanical assessments of stretch‐shortening cycle (SSC) movements such as the countermovement jump (CMJ) are used to evaluate neuromuscular function in alpine ski racers after anterior cruciate ligament reconstruction (ACLR). However, this analysis yields multiple CMJ force‐time metrics that quantify SSC mechanics, creating challenges for data synthesis, interpretation, and return‐to‐sport decision making. Machine learning (ML) classification algorithms address this problem by determining patterns that distinguish healthy control athletes and athletes recovering from ACLR. ML classification algorithms were trained using CMJ force‐time metrics obtained from healthy control elite alpine ski racers (Control) and skiers tested after ACLR to identify features predictive of group membership. Participants (ACLR: *n* = 24, Control: *n* = 42) performed multiple CMJ testing sessions as part of a longitudinal athlete monitoring program (*n* = 836). ML algorithms (random forest, support vector machine, logistic regression, naïve Bayes, *k*‐nearest neighbors) were trained using 23 CMJ force‐time features with 5‐fold cross‐validation and evaluated using an independent test dataset. Classification performance was high with balanced accuracies ranging from 0.59 to 0.88 and areas under the receiver operating characteristic curve of 0.63–0.95. Features corresponding to the propulsion phase were most important for differentiating CMJ tests from ACLR and Control athletes. Recovery of neuromuscular function after ACLR may be inferred when the CMJ mechanics of athletes with ACLR become indistinguishable from those of healthy controls. In conclusion, ML classification models may assist interpretation of CMJ force‐time metrics after ACLR by identifying high‐information features related to injury status along with a potential indication of rehabilitation progression relative to healthy control athletes.

## 1 Introduction

Anterior cruciate ligament (ACL) injuries are traumatic knee injuries occurring frequently in field sports [1, 2, 3, 4] and winter slope sports such as alpine ski racing [5, 6, 7]. In fact, the risk of traumatic knee injury amongst alpine ski racers is exceptionally high and ACL injury is the most frequent and specific diagnosis [8]. Surgical reconstruction of the anterior cruciate ligament (ACLR) is typically recommended in an athlete population [9] and elite alpine ski racers [10] to restore knee joint stability; however, race‐performance deficits [11] and neuromuscular deficits assessed during stretch‐shortening cycle (SSC) movements such as the countermovement jump (CMJ) can persist for up to 3–5 years despite an athlete's return to sport [12, 13]. Further, there is a significant risk of subsequent ACL injury (including to the contralateral limb) in ski racers, which often occurs ~24 months after the index surgery [14, 15, 16].

Thus, biomechanical assessments, including CMJ testing on a dual force plate system, are recommended to determine return‐to‐sport readiness after ACLR and to monitor neuromuscular function in athletes with ACLR as a component of a longitudinal athlete monitoring program [17, 18, 19, 20]. A CMJ force‐time assessment is ideal in this context given it is a non‐fatiguing, practical, and time efficient test that can be used to quantify SSC mechanics and lower limb power in athletes, which provides high relevance for a range of sport‐specific maneuvers involving coupled eccentric‐concentric muscle actions [21]. For example, CMJ force‐time analysis can be used to quantify SSC mechanics during the eccentric deceleration (i.e., braking) and concentric (i.e., propulsion) phases [22], and when combined with a dual force plate system, CMJ testing has been used to quantify interlimb force‐time asymmetries in athletes recovering from ACLR [19, 23, 24, 25]. CMJ testing using a dual force plate system has become increasingly common in athletes with ACLR, with multiple factors influencing the recovery of interlimb force‐time asymmetry such as the graft type (e.g., bone patellar tendon versus semitendinosus autografts) [26] and self‐reported musculoskeletal function [27]. Yet, the interlimb asymmetry index based on the contralateral limb benchmark has been shown to overestimate neuromuscular function in athletes with ACLR [12, 28]. Consequently, recent evidence emphasizes the assessment and monitoring of multiple CMJ force‐time variables that represent distinct constructs related to an athlete's SSC mechanics and power, including those related to CMJ performance (e.g., jump height, peak external mechanical power) [29], CMJ movement strategy (e.g., CMJ contraction time, lower limb stiffness) [17], and interlimb force‐time asymmetry [30], as these variables may demonstrate asynchronous recovery trajectories following ACLR [12].

However, the large number of potentially relevant force‐time variables derived from biomechanical assessments of the CMJ test presents a challenge for clinicians and practitioners to determine the measures that are most sensitive to the post‐ACLR recovery process and to synthesize data in accordance with return‐to‐sport decision making [31, 32]. To address this problem, data‐driven approaches, including machine learning (ML) classification algorithms, may be useful for identifying CMJ force‐time variables most strongly associated with ACL injury status due to their ability to handle complex and multi‐dimensional data [33]. For example, ML models have been developed to assist clinical decision‐making whereby patient characteristics (e.g., health‐, movement‐, performance‐related variables) are used to detect pathology, assess treatment progress, and estimate current and future health outcomes [34, 35, 36, 37]. ML classification algorithms are also useful for assessing the likeness of an observation in relation to predefined categories such as injury status. In this context, Richter et al. applied ML classification algorithms to motion‐capture‐derived time‐series jump data to differentiate between male field sport athletes with ACLR and healthy control athletes [38]. Whether similar ML classification algorithms can be applied to multi‐dimensional CMJ force‐time datasets to determine injury status in healthy control elite alpine ski racers and skiers with ACLR has yet to be explored.

Therefore, the purpose of this study was to investigate and compare the classification accuracy of different ML models trained on CMJ force‐time metrics obtained from healthy control elite alpine ski racers along with skiers with ACLR who performed multiple CMJ testing sessions as a component of a longitudinal athlete monitoring program. A second aim was to identify which CMJ force‐time variables were most important for differentiating ski racers with ACLR from healthy control skiers.

## 2 Methods

### 2.1 Guidelines

The results of this study are reported in accordance with the Transparent Reporting of a multivariable prediction model for Individual Prognosis or Diagnosis (TRIPOD) guidelines [39].

### 2.2 Participants

Elite alpine ski racers who were post‐ACLR (*n* = 24; females: *n* = 14) and healthy control ski racers matched for competition level (*n* = 42; females: *n* = 17) performed multiple CMJ testing sessions on a dual force plate system in their training environment as a component of a longitudinal athlete monitoring program. In total, there were 836 CMJ testing sessions. CMJ testing sessions were performed at a mean post‐operative time of 14 ± 8 months (range: 3–35 months) and the median number of jump assessments per athlete was *n* = 6 for the ACLR group and *n* = 9 for the Control group (Table 1). Participants from the ACLR group and Control group were excluded from the analysis if they were being treated for an injury at the time of testing that prevented maximal effort jumping or had a previous lower limb injury other than ACLR requiring surgery (e.g., leg fracture). Informed consent was provided by all participants, and the study protocols were approved by the University of Calgary Conjoint Health and Research Ethics Board (REB15‐1094).

**TABLE 1:** Countermovement jump test distribution amongst participants.

Status	Total number of athletes	Total number of jump tests	Mean number of jump tests	Median number of jump tests
ACLR	24	146	6.1 ± 4.9	6
Control	42	691	16.5 ± 19.5	9

### 2.3 Countermovement Jump Protocol

Participants performed a standardized warmup consisting of 10 min of cycling on a cycle ergometer and dynamic stretching [12, 23, 24]. All participants were familiarized with the CMJ testing protocol that included 5 maximal effort CMJ repetitions separated by 3 s of quiet standing on the force plates. Participants were instructed to perform the CMJ repetitions with the hands placed firmly on the hips and a cue was given to maximize the vertical jump height using a self‐determined countermovement depth [12, 23, 24]. CMJ repetitions that did not follow the study protocol were discarded and repeated.

### 2.4 Countermovement Jump Force‐Time Analysis

The vertical ground reaction forces (*F*_*z*_) from the left and right limbs were measured simultaneously using a dual force plate system (Accupower Force Platform, AMTI, Watertown, Massachusetts, USA) sampling at 1500 Hz. Data were exported and analyzed using a custom‐built computer program (MATLAB R 2022a, MathWorks, Natwick, MA, USA). The force‐time analysis methodology has been described elsewhere in detail [12, 22, 23, 24]. Briefly, *F*_*z*_ from the left and right limbs were summed to obtain the total *F*_*z*_ for the body center of mass (BCM). Next, the vertical acceleration (*a*_*z*_) was determined using the following equation:

az=Fzbody mass−9.81m/s2

**FIGURE 1:**
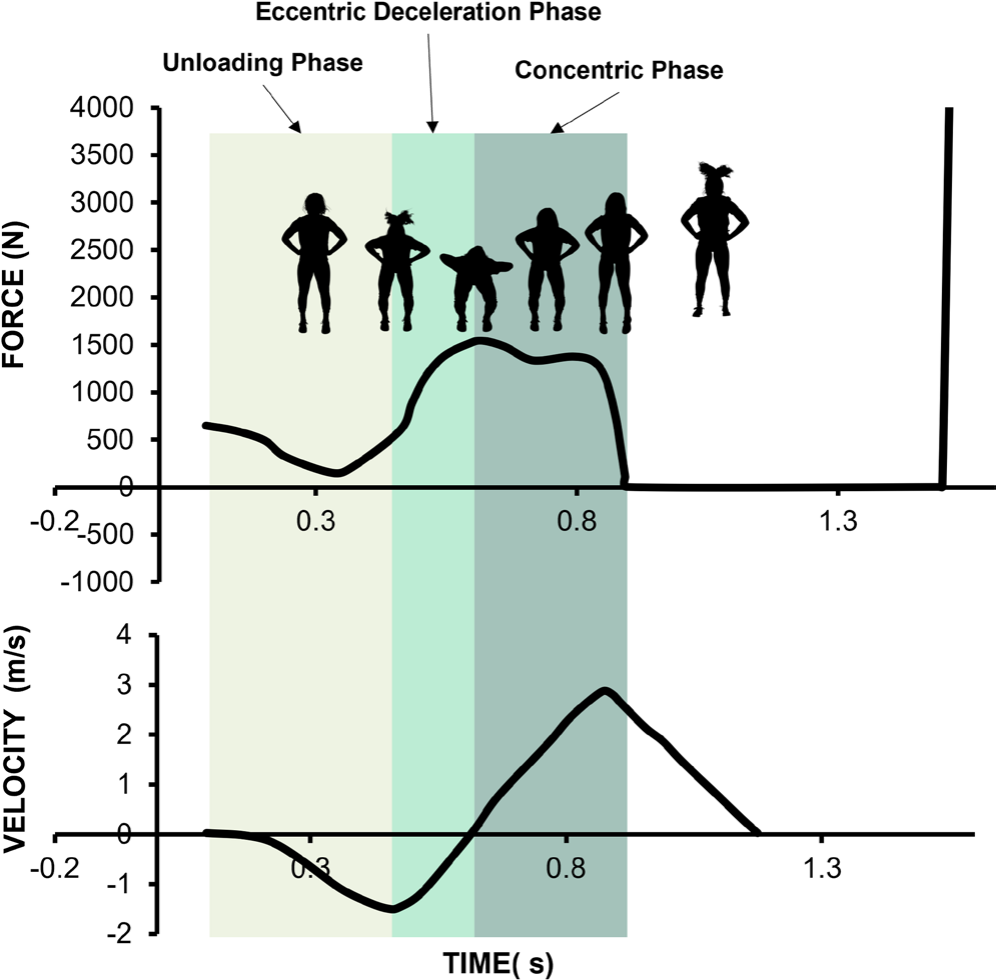
Schematic representation of countermovement jump (CMJ) phases derived from vertical ground reaction force‐time data, including the unloading, eccentric deceleration (braking), concentric (propulsion), and landing phases.

The *a*_*z*_ curve was integrated with respect to time to obtain the velocity of the BCM (*v*_*z*_). The eccentric deceleration phase was defined as the time interval from the maximum downward negative *v*_*z*_ to the instant the BCM *v*_*z*_ was equal to zero, corresponding to the lowest position of the CMJ (Figure 1). The concentric (propulsion) phase was determined from this time point to the instant of takeoff (Figure 1). The net eccentric deceleration impulse was obtained by time integration *F*_*z*_ over the appropriate time interval and jump height was obtained from *v*_*z*_ at takeoff using the following equation:

$$\text{Jump height}=\frac{{\left(v_{z\;\mathrm{at}\;\text{takeoff}}\right)}^{2}}{2g}$$

The CMJ contraction time was calculated from the onset of the countermovement to the instant the athlete left the force plate at toe‐off. This allowed the reactive strength ratio (RSR) to be calculated using the predicted flight time of the BCM obtained from the jump height divided by the CMJ contraction time using the following equations:

$$\text{Predicted flight time of the}\;\mathrm{BCM}=\sqrt{\frac{\text{Jump height}\times 8}{g}},$$

$$\mathrm{RSR}=\frac{\text{Predicted flight time of the}\;\mathrm{BCM}}{\mathrm{CMJ}\;\text{contraction time}}$$

The RSR metric is expressed as a unitless ratio consistent with the broader force‐time analytical framework [12].

The external mechanical power generated in the CMJ was calculated as the instantaneous product of *F*_*z*_ and *v*_*z*_ ($\text{External mechanical power}=F_{z}\cdot v_{z}$) and the peak propulsive power and peak braking power were obtained along with the *F*_*z*_ and *v*_*z*_ at peak power [22]. The *F*_*z*_ at maximum *v*_*z*_ was also obtained [40]. Finally, lower limb stiffness (LL_STIFF_) was calculated over the eccentric deceleration phase as:

$${\mathrm{LL}}_{\text{STIFF}}=\frac{∆F_{Z}}{∆\text{Downward displacement of the}\;\mathrm{BCM}}$$

### 2.5 Countermovement Jump Force‐Time Interlimb Asymmetry Index Calculation

The left and right limb eccentric deceleration impulses and concentric impulses were obtained by time integration of *F*_*z*_ over the respective time intervals. Interlimb asymmetry indexes (AI%) were calculated for the eccentric deceleration phase and concentric phase as:

$$\begin{array}{c} \begin{array}{c} \text{ACLR group}\;\mathrm{AI}\left(\%\right)=\frac{\text{Unaffected limb}\;-\;\text{Affected limb}}{\text{Maximum of the unaffected}\;\mathrm{vs}.\;\text{affected limb}}\times 100 \end{array} \end{array}$$

$$\text{Control group}\;\mathrm{AI}\left(\%\right)=\frac{\text{Right limb}-\text{Left limb}}{\text{Maximum of the right}\;\mathrm{vs}.\;\text{left limb}}\times 100$$

### 2.6 Variable Selection

A five‐jump mean value for the CMJ force‐time metrics and the maximum jump height were obtained for the analysis. Force‐ and power‐based outcome measures were body mass normalized. The CMJ force‐time metrics have been previously examined for reliability [12]. In total, 23 CMJ force‐time metrics were included as features in the analysis that are described in detail in Table 2. Variables were selected based on their previous utility for assessing mechanical muscle function in various populations, including ACL injured athletes [12, 24], healthy athletes [41], and to evaluate neuromuscular function in younger and older adults [42]. Continuous variables were standardized using *z*‐score normalization, with scaling parameters derived exclusively from the training data and applied to the independent test set to prevent information leakage. The values were normalized using the scale function in R. (R, Version 4.2.3).

**TABLE 2:** Description of countermovement jump variables included in the models.

Variable	Abbreviation	Description
Jump height	JH	Maximum jump height achieved
Takeoff velocity	Vtakeoff	Velocity at the point of takeoff
Peak external mechanical power	PP	Maximum value of the external mechanical power generated relative to body mass
Rate of power development	RPD	Average increase in power during the concentric phase
Force at maximal velocity	FzVmax	The force at the time point where maximal velocity occurs
Force at zero velocity	FzVzero	The force exerted at the lowest point of the jump during the transition from eccentric to concentric movement
Net concentric impulse	ConImp	The impulse during the concentric phase divided by body mass
Net eccentric deceleration impulse	EccImp	The impulse during the eccentric deceleration phase divided by body mass
Net unloading impulse	UnImp	The impulse during the unloading phase divided by body mass
Minimum power (peak braking power)	Pmin	Peak negative value of the power‐time curve
Minimum velocity (peak braking velocity)	Vmin	Peak negative value of the body center of mass velocity
Time to peak power	TTPP	Time taken to reach peak power
Velocity at peak power	VPP	The velocity at the time point where peak power occurs
Force at peak power	FzPP	The force at the time point where peak power occurs
Contraction time	TotDur	Time taken to perform the entire CMJ
Concentric phase duration	ConDur	Time taken to perform the concentric phase
Eccentric phase duration	EccDur	Time taken to perform the eccentric deceleration phase
Lower limb stiffness	LLS	The change in force divided by the change in displacement of the body center of mass during the eccentric deceleration phase
Concentric phase impulse asymmetry index	ConAI	Between‐limb impulse asymmetry during the concentric phase
Eccentric phase impulse asymmetry index	EccAI	Between‐limb impulse asymmetry during the eccentric deceleration phase
Landing phase impulse asymmetry index	LandAI	Between‐limb impulse asymmetry during the landing phase
Reactive strength ratio	RSR	Predicted flight time/contraction time
Maximal velocity—takeoff velocity difference	Vmax–Vtakeoff	The difference between maximal velocity and takeoff velocity

### 2.7 Model Development

The unit of analysis for all models was the individual CMJ testing session rather than the athlete. Because athletes contributed repeated observations, group cross‐validation was implemented at the athlete level to account for non‐independence and avoid inflation of model performance estimates. Accordingly, model performance metrics reflect classification at the CMJ test level rather than the athlete level and should be interpreted within this context. Athletes were grouped into a training cohort (ACLR, *n* = 16, jumps = 95; Control, *n* = 30, jumps = 526) and a test cohort (ACLR, *n* = 8, jumps = 51; Control, *n* = 12, jumps = 164) using a 70:30 random split. The training cohort was used to train 5 unique machine learning algorithms: random forests, support vector machines (SVMs), logistic regression, *k*‐nearest neighbors (KNN), and naïve Bayes.

These classifiers are commonly used in injury forecasting [43] and have been used previously with biomechanical assessments for differentiating between healthy control athletes and ACLR athletes [38]. The CMJ tests in the training cohort were partitioned into five folds, using a grouped cross‐validation approach, ensuring that all CMJ tests from a given athlete were confined to the same fold. This approach prevented any data leakage due to repeated measures from the same athlete appearing in both training and validation sets. Five‐fold cross‐validation was used to assess model performance and, where applicable, a grid search was used for hyperparameter tuning. For random forest, the following parameters were tested with a 3 × 3 grid search: number of trees: {100, 500, 1000}; variables considered at each split {2, 4, 6}. For SVM, a 4 × 4 grid search of the following parameters were tested: cost: {0.1, 1, 10, 100}; gamma {0.001, 0.01, 0.1, 1}. For KNN, *k* values of 1 to 20 were tested in steps of 1. The hyperparameters resulting in the greatest accuracy across each of the validation sets were then used in the experiments with the test cohort to compute final evaluation metrics. The hyperparameters used in the experiments with the test set were: number of trees = 1000, variables considered at each split = 6, cost = 10, gamma = 0.01, *k* = 1.

### 2.8 Assessment of Model Performance

The performance of each algorithm was evaluated through the area under the receiver operating characteristic (AUROC) which varies from 0.5 (random predictor) to 1 (perfect predictor). Performance was also evaluated using accuracy ($\frac{\text{true positives}+\text{true negatives}}{\text{total pedictions}}$), precision ($\frac{\text{true positives}}{\text{true positives}+\text{false positives}}$), recall ($\frac{\text{true positives}}{\text{true positives}+\text{false negatives}}$), *F*‐1 score ($\frac{\text{precision}+\text{recall}}{2}$), and balanced accuracy ($\frac{\text{recall}+\text{specificity}}{2}$).

The importance of each variable included in the random forest was assessed using permutation importance, otherwise known as mean decrease in accuracy, which measures the decrease in model accuracy when a particular variable is permuted [44, 45].

### 2.9 Quantifying the Time‐Course Change in the Probability of Group Membership

A feature of the ML methodology used in the present study is the ability to generate a predicted probability of group membership at the individual level. Thus, to explore the utility of this feature in the context of ACLR rehabilitation, the predicted probabilities of CMJ testing sessions belonging to the healthy control group were calculated over time for three participants from the ACLR group. Predicted probabilities were obtained from the random forest model and represent the proportion of decision trees voting for a given group. Predicted probabilities corresponding to classification to the healthy control group were extracted and plotted as a function of time since surgery. A loess smoothing function was applied to visualize trajectories, and a decision boundary separating ACLR versus healthy control group classification was set at a probability threshold of 0.50. Analyses were performed in RStudio using various open‐source packages including the randomForest, e1071, class, and pROC packages (R, Version 4.2.3).

## 3 Results

Performance metrics were obtained from model training and independent testing. From our training cohort, we identified the highest accuracy achieved during cross‐validation across each of the five folds and calculated the average accuracy. The resulting average accuracies (±SD) were 88% ± 4% for random forest, 88% ± 4% for SVM, 82% ± 1% for logistic regression, 77% ± 5% for naïve Bayes, and 84% ± 5% for KNN.

**FIGURE 2:**
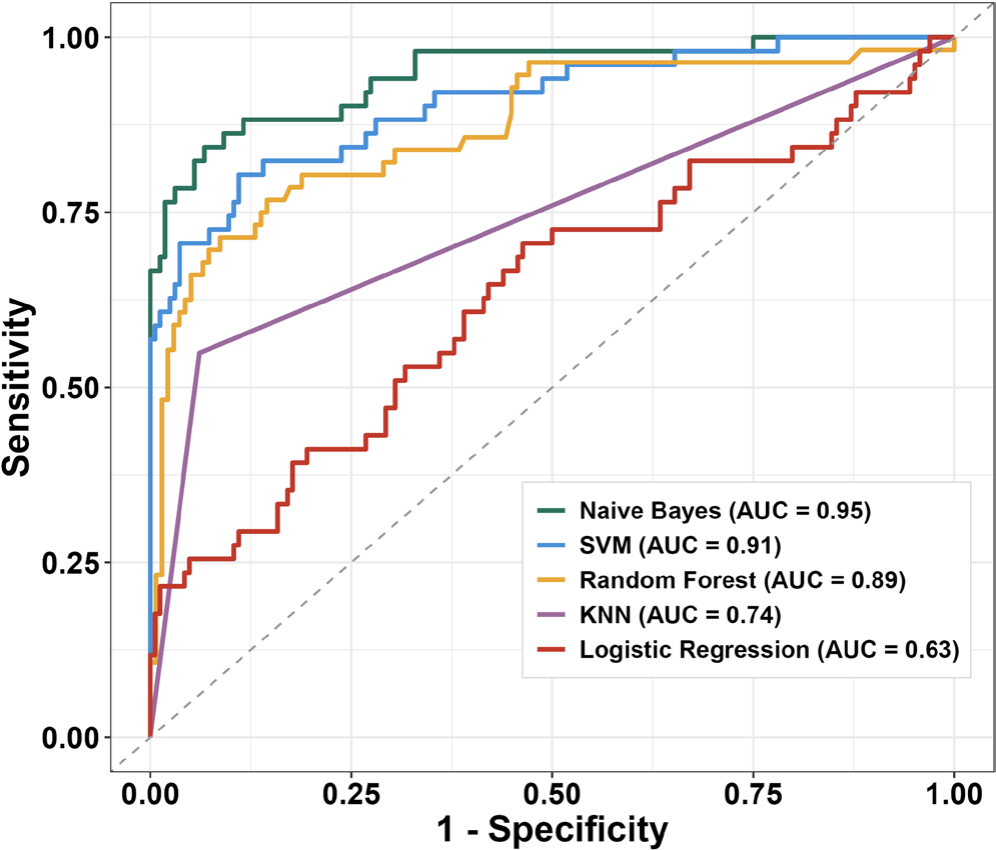
Receiver operating characteristic curves for models applied to countermovement jump tests from the test cohort (*n* = 215); AUC, area under the curve; KNN, k nearest neighbor; SVM, support vector machine.

The performances of the 5 models on the independent test cohort are displayed in Table 3. The best performing models were the naïve Bayes and the random forest with accuracies of 87% and 89% and AUROC's of 0.95 and 0.89 respectively (Figure 2).

**TABLE 3:** Model performance during independent testing of countermovement jumps (*n* = 215).

Metric	Random forest	Support vector machine	Logistic regression	Naïve Bayes	*k*‐nearest neighbors
Area under the ROC curve	0.885	0.907	0.633	0.947^a^	0.744
Accuracy	0.893^a^	0.865	0.739	0.874	0.847
Precision	0.850	1.00^a^	0.429	0.681	0.737
Recall	0.667	0.431	0.294	0.882^a^	0.549
*F*1 score	0.747	0.603	0.349	0.769^a^	0.629
Balanced accuracy	0.815	0.716	0.586	0.877^a^	0.744

^a^ Best performing model.

**FIGURE 3:**
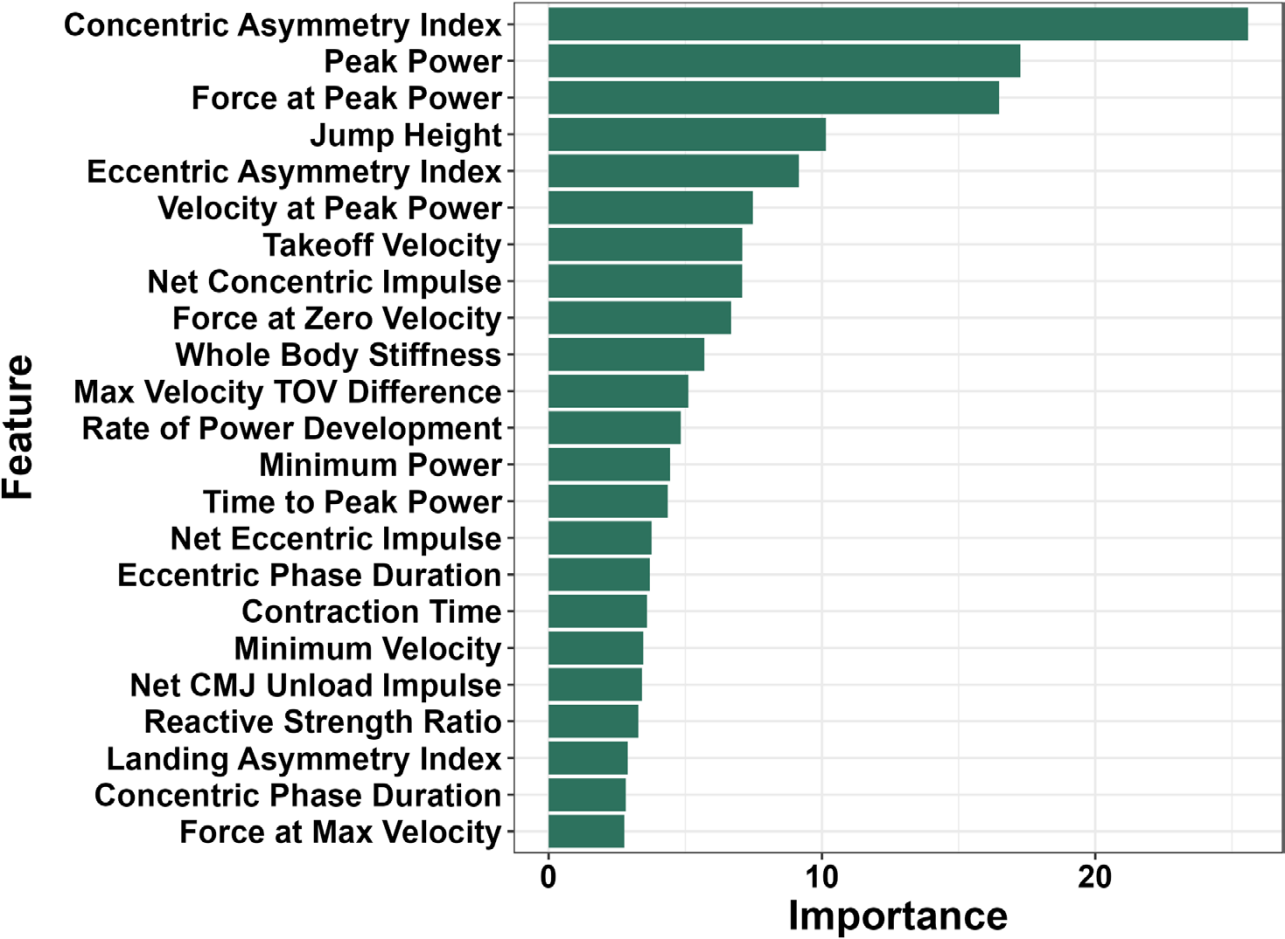
Variable importance plot from the random forest model applied to the independent test cohort. Higher values indicate greater contribution to distinguishing countermovement jump tests from athletes with ACL reconstruction and healthy control athletes, with concentric‐phase variables demonstrating the greatest relative importance.

The most important variables indicated by the random forest were the concentric phase impulse asymmetry index, peak external mechanical power, force at peak power, jump height, and the eccentric phase impulse asymmetry index. The least important variables were the landing phase impulse asymmetry index, concentric phase duration, and force at maximum velocity (Figure 3).

**FIGURE 4:**
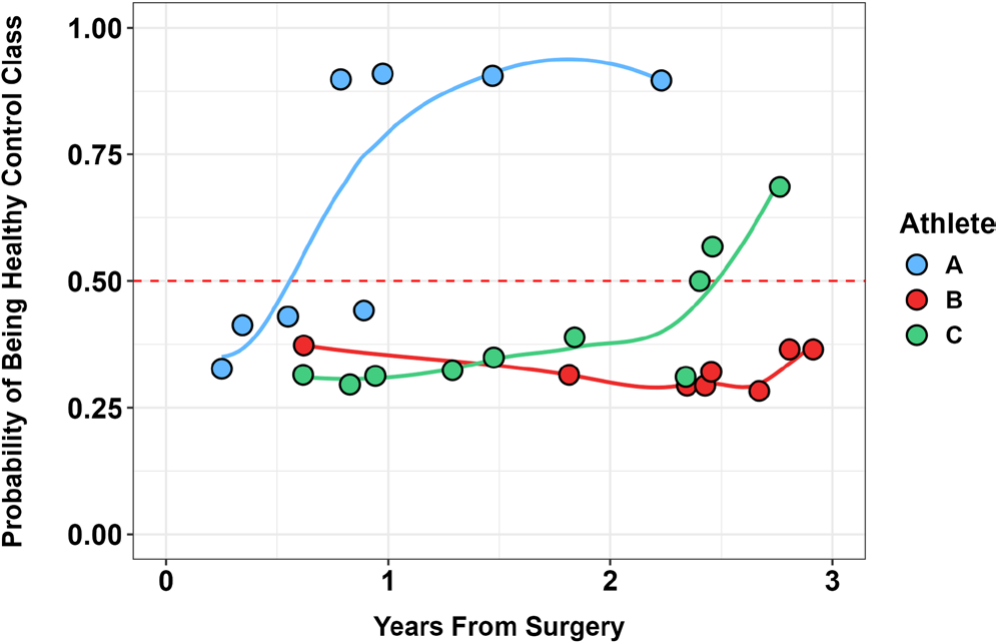
The time‐dependant probability of healthy control group classification for countermovement jump (CMJ) tests of three ski racers post‐surgery using the random forest model. The dots represent the individual probability predictions over time, reflecting the proportion of trees that classified the CMJ test as belonging to the healthy control group. The solid lines represent the smoothed trends (via loess) of these probabilities. The red dashed horizontal line represents the threshold at which the model is equally likely to classify a CMJ test as being either from the ACLR group or the healthy control group.

The predicted probabilities from the random forest model plotted over time are shown in Figure 4. Three contrasting time‐course changes in the predicted probabilities are provided (Figure 4). The CMJ testing from Athlete A demonstrated a high probability of healthy control group membership at ~12 months post‐ACLR (probability > 0.95). This is contrasted with Athlete B, whose CMJ testing sessions were still below the decision boundary at 3 years post‐ACLR (probability < 0.5). Finally, the CMJ testing sessions of Athlete C demonstrated a probability of healthy control group membership above the decision boundary at ~2.5 years post‐ACLR. Notably, the predicted probability of group membership represents a weighted combination of CMJ features based on interlimb force‐time asymmetries, CMJ performance, and CMJ movement strategy metrics. A high probability of healthy control group membership may be considered as the participant's CMJ testing session biomechanics being indistinguishable from those of the healthy control group.

## 4 Discussion

This study provides new insights on how ML classification algorithms can be combined with longitudinal biomechanical assessments of CMJ SSC movements using a dual force plate system to evaluate elite high‐performance athletes after ACL injury. We tested and compared the accuracy of five classification algorithms trained on CMJ force‐time metrics obtained from healthy control elite ski racers and ski racers with ACLR across multiple CMJ testing sessions as a part of a longitudinal athlete monitoring program. On average, the five algorithms produced classification accuracies of 84% and balanced accuracies of 75%, suggesting that the CMJ force‐time metrics contained sufficient information to distinguish between the CMJ tests from healthy control ski racers and those with ACLR. Further, CMJ force‐time variables related to the propulsion phase, notably the concentric impulse asymmetry index, the peak external mechanical power, the force produced at peak power, and the CMJ height along with the eccentric deceleration impulse asymmetry index, were of the highest feature importance.

**FIGURE 5:**
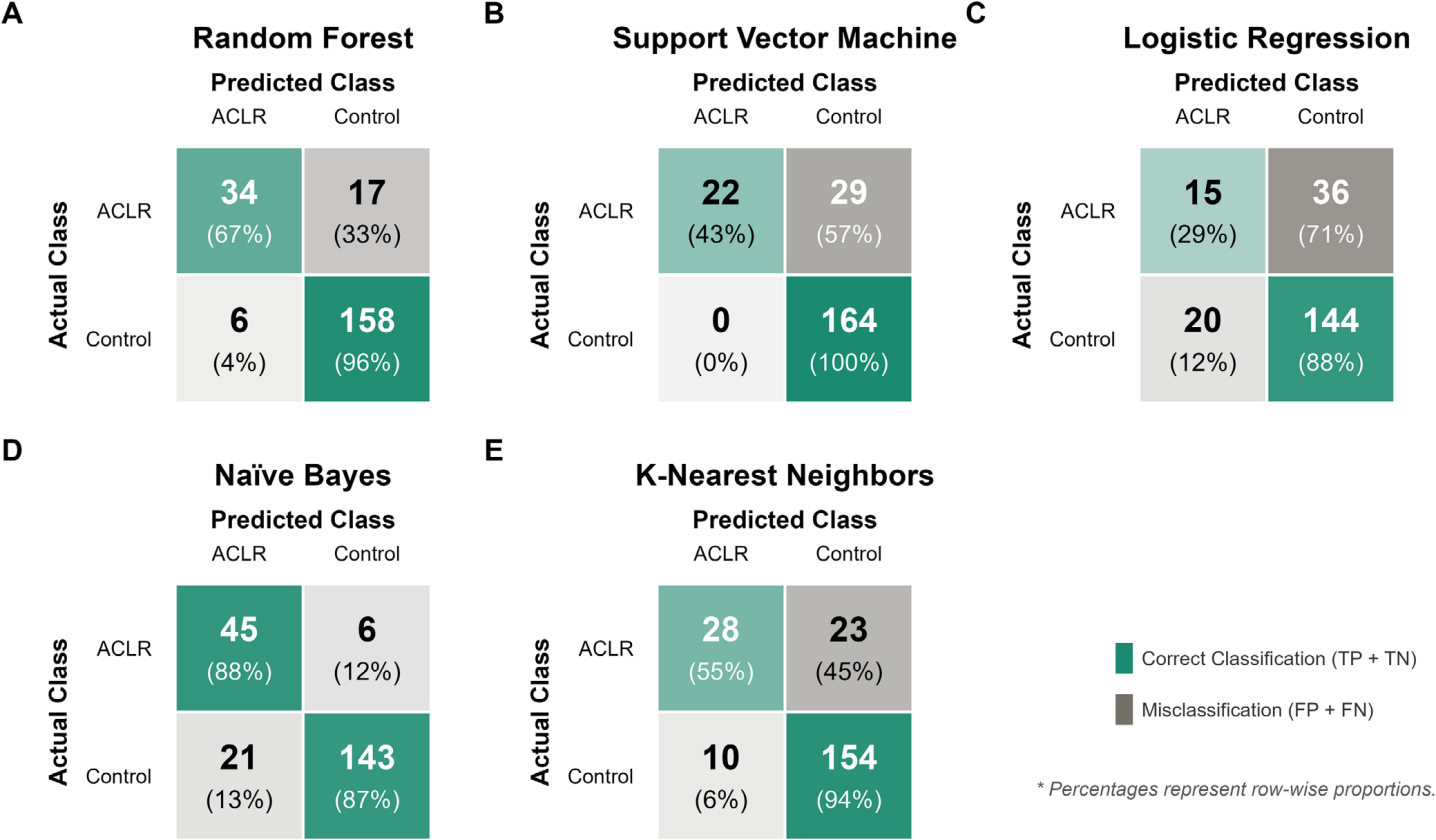
Confusion matrix for models applied to the countermovement jump (CMJ) tests of the independent test cohort. FN, false negative; FP, false positive; TN, true negative; TP, true positive. The matrices illustrate high control classification rates with lower sensitivity for CMJ tests from ACLR athletes, indicating that some CMJ tests from ACLR athletes were classified as being from healthy control athletes and therefore exhibited biomechanical patterns that were less distinguishable from healthy control athletes.

The strong model performance may be partially explained by the high specificity observed across models (i.e., a low percentage of CMJ tests from the healthy control ski racers were falsely classified as ACLR). For example, 0 of the 164 CMJ tests from the healthy control group were classed as belonging to the ACLR group with the SVM model and only 5 CMJ tests from the healthy control group were classed as belonging to the ACLR group with the random forest model (Figure 5). This suggests the existence of commonalities in the CMJ force‐time metrics of high‐level healthy control ski racers. Ski racing is a bilateral sport with bidirectional turning and is dominated by high‐force contractions at low velocities and quasi‐isometric loading of the leg extensors [46, 47, 48]. Consistent with the mechanical demands of the sport, healthy elite alpine ski racers have been shown to present with minimal CMJ force‐time interlimb asymmetry [24] and CMJ profiles characterized by higher maximal force and lower velocity capacity compared to sprinters [49]. This relatively homogeneous CMJ mechanical profile amongst healthy control ski racers may reduce within‐group variability and contribute to clearer differentiation between injured and healthy control athletes. Accordingly, ski racers who deviated from this CMJ mechanical profile by displaying large interlimb force‐time asymmetry and reduced CMJ performance may be more likely to be classified as injured.

Figure 5 also suggests that the classifiers considered in our study provide complementary views regarding the predictions. For example, while SVM led to high classification results for the healthy control group, naïve Bayes yielded the best results for the ACLR group. These results suggest the utility of combining different classifiers tailored to the problem at hand as a promising area of future research [50]. Despite the high specificity observed, the unequal distribution of CMJ testing sessions between groups may have introduced the potential for group imbalance to influence performance metrics. Accuracy alone may therefore overestimate model performance by favoring the healthy control group, which was relatively overrepresented compared to the ACLR group. Balanced accuracy was utilized to ensure the evaluation remained robust to group imbalance.

Additionally, the variation in precision and recall across models indicates that model interpretation should be aligned with the clinical objective, particularly when considering the consequences of false positive versus false negative classifications during rehabilitation decision‐making. Consequently, models such as the random forest and SVM, which demonstrated a more favorable balance between sensitivity and specificity than naïve Bayes, may offer greater clinical utility for identifying persistent functional deficits.

It was notable that classification errors mainly occurred through false negatives in the ACLR group where a given CMJ test was classified as belonging to the healthy control group. An alternative interpretation is that these false negatives for the ACLR group corresponded to CMJ tests that were indistinguishable from the healthy control group. Thus, such a finding may indicate that the CMJ force‐time profile reached the level of the healthy control group providing a potential indication of an individual's rehabilitation status. Interestingly, when we examined the CMJ testing sessions misclassified as belonging to the healthy control group from the random forest model, the concentric impulse asymmetry index and eccentric deceleration impulse asymmetry index were less than 10%, which is often recommended as a target for interlimb asymmetry prior to return to sport [29, 51]. However, moving beyond measures of interlimb force‐time asymmetry that are known to potentially overestimate rehabilitation status in an athlete population with ACLR [12, 52], the present finding should be interpreted as a wholistic CMJ force‐time profile based on the ML model, which combined measures of interlimb force‐time asymmetry, CMJ performance and the CMJ movement strategy. Therefore, these results may be favored over the interpretation of isolated CMJ force‐time metrics or interlimb asymmetry values since the ML model represents a weighted combination of CMJ force‐time and performance characteristics (i.e., a CMJ mechanical profile) using a complex systems approach. This complex systems approach to data analytics represents a promising area for future research in sport science and sport medicine practice [53].

There is limited research using biomechanical data to classify membership to a particular group (e.g., healthy control vs. injured, elite vs. sub‐elite). In one of the few related studies, Richter et al., collected biomechanical data of various jump and change of direction movements from ACLR athletes and healthy control athletes and used a non‐biased feature extraction procedure and various supervised ML techniques to differentiate the groups [54]. In their study, for the bilateral CMJ, the logistic regression was the best performing model with an accuracy of 73% followed closely by the NB and neural network. This is comparable to the balanced accuracy findings across the 5 models used in our study. However, in a follow up cross‐validation of their model on female athletes from different sporting populations and using a different CMJ testing protocol, the ability to identify athletes with previous ACLR was poor [37], illustrating the importance for out‐of‐sample validation before implementation.

The reporting of feature importance in our study is a novel contribution to the literature that can help practitioners and clinicians prioritize the multitude of CMJ mechanical variables obtained when employing a dual force plate system to evaluate SSC function in athletes. The models developed in this study used 23 features, including CMJ force‐time metrics that are typically derived and monitored following ACLR, such as the impulse asymmetry index, reactive strength ratio (RSR), jump height, and peak external mechanical power [17, 26] in addition to other mechanical variables, such as force at zero velocity, rate of power development, and lower limb stiffness [12, 41, 42, 55]. In our study, the features with the greatest importance to the classification performance of our random forest model were the concentric impulse asymmetry index, peak external mechanical power, jump height, the force at peak power, and the eccentric deceleration impulse asymmetry index. Notably, these features are mostly related to the propulsion phase of the CMJ movement, which suggests that impairments in concentric muscular actions are the key factors separating healthy control athletes from those with ACLR. Features specific to the unloading phase, the landing phase, and time‐domain measures (CMJ contraction time, RSR, eccentric deceleration phase duration) were not as important for differentiating classes.

These findings are aligned with recent CMJ analyses from other sporting populations. For example, Kotsifaki et al. assessed the CMJ in 126 professional and recreational athletes with ACLR following return to sport and found significant impulse asymmetry in the concentric phase but not the eccentric phase. A subgroup comparison of the professional soccer athletes (ACLR = 94, Control = 532) showed significant deficits in jump height for the ACLR group [56]. Similarly, Read et al., used a logistic regression analysis on 370 male professional soccer players to identify the CMJ variables that are most strongly associated with a history of ACLR and found the concentric impulse asymmetry index to be the strongest predictor of group classification, followed by jump height and peak landing force of the affected limb [57].

Graft type may influence phase‐specific deficits in the CMJ, as athletes receiving bone‐patellar tendon‐bone autografts have displayed higher eccentric deceleration phase force‐time asymmetry compared to those who received hamstring tendon autografts [26]. In our study, 21 of 24 ski racers received hamstring tendon autografts. Because graft type was not included as a model variable, graft‐related differences may have influenced feature importance and should be considered a potential confounder when interpreting the relative contribution of eccentric versus concentric phase variables.

### 4.1 Practical Applications

Current return‐to‐sport frameworks recommend that athletes with ACLR pass objectively determined return‐to‐sport criteria from a multi‐modal battery of neuromuscular tests [18, 51, 58]. Yet, the ability of current testing batteries to differentiate those who are sufficiently rehabilitated and therefore at reduced risk of subsequent ACL injury and those who have a higher likelihood of restoring performance is poor [52, 59]. Although our classification models were based only on CMJ force‐time assessments, they demonstrated high classification accuracies and specificities within this cohort of athletes. These findings suggest that CMJ force‐time characteristics contain distinct information capable of differentiating athletes with ACLR from healthy control athletes within a homogeneous sporting population. If these assumptions are true, ML may offer an objective approach to classify an athlete's biomechanical patterns and identify the features most strongly associated with group differentiation. However, the clinical significance of being classified as exhibiting a healthy control CMJ force‐time profile is unclear, and future prospective work is needed to determine whether classification is associated with a reduced risk of subsequent ACL injury or performance outcomes.

A potential application of this concept is provided in Figure 4 using model‐derived predictive probabilities for classification to the healthy control group. The predictive probabilities reflect the probability that a CMJ testing session of an athlete with ACLR belonged to the healthy control group. To illustrate a contrasting interpretation of such a model output, the time‐course probabilities of healthy control group classification were plotted for three athletes with ACLR. For Athlete A, the probability of healthy control group membership was > 0.95 at ~12 months post‐surgery compared to Athletes B and C whose predicted probabilities remained below the decision boundary.

Importantly, these probabilities reflect the integrated influence of multiple CMJ force‐time metrics considered simultaneously by the model, including variables related to interlimb force‐time asymmetry, CMJ performance, and CMJ movement strategy, rather than single metrics interpreted in isolation. Broadly, the results presented in Figure 4 demonstrate the heterogeneous and dynamic time‐course change of the CMJ force‐time biomechanics in relation to the healthy control group. A high predicted probability that a CMJ testing session belonged to the healthy control group may also be interpreted as the CMJ biomechanics of the injured athlete being indistinguishable from the CMJ biomechanics of healthy control athletes. Whether this is a valid approach for quantifying rehabilitation status and has bearing on return to performance outcomes or subsequent ACL injury risk is entirely speculative. However, future research may consider comparing this approach with conventional testing methodologies based on the limb symmetry index or asymmetry index using the contralateral limb benchmark.

### 4.2 Limitations

The features used in the ML models were included based on previously identified CMJ force‐time metrics in the literature used to quantify SSC mechanics based on expert consensus and reports in the scientific literature [21, 54]. Nonetheless, discrete CMJ force‐time metrics from traditional analyses do not capture all of the information within the entire kinetic waveform and potentially valuable information may be discarded [60]. Further, force variables were not allometrically scaled, and body mass normalization was used instead of fat‐free‐mass normalization, which may be penalizing, especially for female athletes [61, 62]. Therefore, some features may partially reflect anthropometric differences rather than differences in neuromuscular function. However, the stability of model performance following body mass normalization suggests that this influence was likely minimal. Additionally, we did not employ any feature selection techniques, possibly leading to an overfitting effect, and a reduced feature model may have performed similarly or better [38]. As suggested by Richter et al. [37] classification models using a variety of movement tasks that combine expert‐knowledge‐driven and data‐driven feature extraction techniques are likely to perform better and identify more relevant features for distinguishing classes. Finally, there was a sex imbalance in the cohort, and sex‐specific biomechanical differences were not explored. As such, sex‐related effects may have influenced classification performance and could limit the generalizability of the findings across male and female athletes.

## 5 Perspective

This study contributes new knowledge on how assessing CMJ SSC mechanics with a dual force plate system may help characterize neuromuscular function following ACLR in an elite athlete population. We observed good predictive performance (up to 89% accuracy) of supervised ML models trained to classify CMJ force‐time tests of healthy control ski racers and those with ACLR. We also provide valuable information to clinicians and practitioners by presenting which of the CMJ force‐time metrics were most important for classification. Variables that were specific to the CMJ concentric or propulsion phase and the interlimb impulse asymmetry indexes were the most important features for classification whereas time‐domain measures and force‐time measures corresponding to the unloading and landing phases were not as important.

Given the limitations of currently recommended functional tests in return‐to‐sport decision making after ACLR [52, 59], this study highlights the potential utility of ML approaches for supporting clinician and practitioner evaluations. These findings suggest that routinely collected CMJ force‐time data may provide interpretable insight into neuromuscular function. However, CMJ force‐time test classification as belonging to a healthy control group should not be interpreted as evidence of readiness for return to sport, as return‐to‐sport decisions are multifactorial and extend beyond performance in a single movement or task. Future research should consider training these models on larger datasets, incorporating other neuromuscular, sport‐specific, and psychological measures relevant to functional recovery following ACLR.

## Funding

The authors have nothing to report.

## Conflicts of Interest

The authors declare no conflicts of interest.

## Data Availability

The data that support the findings of this study are available on request from the corresponding author. The data are not publicly available due to privacy or ethical restrictions.

## References

[1] C. C. Prodromos , Y. Han , J. Rogowski , B. Joyce , and K. Shi , “A Meta‐Analysis of the Incidence of Anterior Cruciate Ligament Tears as a Function of Gender, Sport, and a Knee Injury‐Reduction Regimen,” Arthroscopy: The Journal of Arthroscopic and Related Surgery 23 (2007): 1320–1325. 18063176 10.1016/j.arthro.2007.07.003

[2] J. T. Bram , L. C. Magee , N. N. Mehta , N. M. Patel , and T. J. Ganley , “Anterior Cruciate Ligament Injury Incidence in Adolescent Athletes: A Systematic Review and Meta‐Analysis,” American Journal of Sports Medicine 49 (2021): 1962–1972. 33090889 10.1177/0363546520959619

[3] M. Waldén , M. Hägglund , H. Magnusson , and J. Ekstrand , “ACL Injuries in Men's Professional Football: A 15‐Year Prospective Study on Time Trends and Return‐To‐Play Rates Reveals Only 65% of Players Still Play at the Top Level 3 Years After ACL Rupture,” British Journal of Sports Medicine 50 (2016): 744–750. 27034129 10.1136/bjsports-2015-095952

[4] C. C. Dodson , E. S. Secrist , S. B. Bhat , D. P. Woods , and P. F. Deluca , “Anterior Cruciate Ligament Injuries in National Football League Athletes From 2010 to 2013: A Descriptive Epidemiology Study,” Orthopaedic Journal of Sports Medicine 4 (2016): 1–5. 10.1177/2325967116631949 PMC4780097 26998501

[5] T. Bere , T. W. Flørenes , L. Nordsletten , and R. Bahr , “Sex Differences in the Risk of Injury in World Cup Alpine Skiers: A 6‐Year Cohort Study,” British Journal of Sports Medicine 48 (2014): 36–40. 23673520 10.1136/bjsports-2013-092206

[6] C. Raschner , H. p. Platzer , C. Patterson , I. Werner , R. Huber , and C. Hildebrandt , “The Relationship Between ACL Injuries and Physical Fi Tness in Young Competitive Ski Racers : A 10‐Year Longitudinal Study,” British Journal of Sports Medicine 46 (2012): 1–7. 10.1136/bjsports-2012-091050 22968156

[7] T. W. Flørenes , T. Bere , L. Nordsletten , S. Heir , and R. Bahr , “Injuries Among Male and Female World Cup Alpine Skiers,” British Journal of Sports Medicine 43 (2009): 973–978. 19945979 10.1136/bjsm.2009.068759

[8] M. Jordan , J. Spörri , and J. Taylor , “Injury Prevention and Rehabilitation,” in The Science of Alpine Ski Racing (Routledge, 2023).

[9] C. L. Ardern , K. E. Webster , N. F. Taylor , and J. A. Feller , “Return to Sport Following Anterior Cruciate Ligament Reconstruction Surgery: A Systematic Review and Meta‐Analysis of the State of Play,” British Journal of Sports Medicine 45 (2011): 596–606. 21398310 10.1136/bjsm.2010.076364

[10] M. J. Jordan , P. Doyle‐Baker , M. Heard , P. Aagaard , and W. Herzog , “A Retrospective Analysis of Concurrent Pathology in ACL‐Reconstructed Knees of Elite Alpine Ski Racers,” Orthopaedic Journal of Sports Medicine 5 (2017): 2325967117714756. 28812037 10.1177/2325967117714756 PMC5528939

[11] N. Morris , R. Da Silva Torres , M. Heard , P. Doyle Baker , W. Herzog , and M. J. Jordan , “Return to On‐Snow Performance in Ski Racing After Anterior Cruciate Ligament Reconstruction,” American Journal of Sports Medicine 53 (2025): 640–648. 39834107 10.1177/03635465241307212 PMC11874593

[12] M. J. Jordan , N. Morris , S. Nimphius , P. Aagaard , and W. Herzog , “Attenuated Lower Limb Stretch‐Shorten‐Cycle Capacity in ACL Injured vs. Non‐Injured Female Alpine Ski Racers: Not Just a Matter of Between‐Limb Asymmetry,” Frontiers in Sports and Active Living 4 (2022): 853701. 35434617 10.3389/fspor.2022.853701 PMC9008592

[13] P. J. Read , D. Cscs , W. T. Davies , et al., “Residual Deficits in Reactive Strength After Anterior Cruciate Ligament Reconstruction in Soccer Players,” Journal of Athletic Training 58 (2023): 423–429. 37523420 10.4085/0169-20 PMC11220905

[14] A. Haida , N. Coulmy , F. Dor , et al., “Return to Sport Among French Alpine Skiers After an Anterior Cruciate Ligament Rupture: Results From 1980 to 2013,” American Journal of Sports Medicine 44 (2016): 324–330. 26598331 10.1177/0363546515612764

[15] C. Nagelli , “Should Return to Sport Be Delayed Until 2 Years After Anterior Cruciate Ligament Reconstruction? Biological and Functional Considerations,” Sports Medicine 47, no. 2 (2016): 221–232. 10.1007/s40279-016-0584-z PMC5226931 27402457

[16] M. J. Jordan , P. Aagaard , and W. Herzog , “Anterior Cruciate Ligament Injury/Reinjury in Alpine Ski Racing: A Narrative Review,” Open Access Journal of Sports Medicine 8 (2017): 71–83. 28435336 10.2147/OAJSM.S106699 PMC5386612

[17] M. J. Jordan , N. Morris , M. Lane , et al., “Monitoring the Return to Sport Transition After ACL Injury: An Alpine Ski Racing Case Study,” Frontiers in Sports and Active Living 2 (2020): 496980. 10.3389/fspor.2020.00012 PMC7739580 33345007

[18] M. Buckthorpe , “Optimising the Late‐Stage Rehabilitation and Return‐To‐Sport Training and Testing Process After ACL Reconstruction,” Sports Medicine 49 (2019): 1043–1058. 31004279 10.1007/s40279-019-01102-z

[19] M. J. Jordan , N. Morris , J. Barnert , D. Lawson , I. Aldrich Witt , and W. Herzog , “Forecasting Neuromuscular Recovery After Anterior Cruciate Ligament Injury: Athlete Recovery Profiles With Generalized Additive Modeling,” Journal of Orthopaedic Research 40 (2022): 2803–2812. 35194823 10.1002/jor.25302 PMC9790559

[20] W. M. J. Jordan , P. Aagaard , C. Bishop , et al., “Explosive Strength and Stretch‐Shortening‐Cycle Capacity During ACL Rehabilitation‐Mechanical Biomarkers for Return to Sport and Performance Readiness,” Aspetar Sports Medicine Journal 12 (2023): 324–331.

[21] C. Bishop , M. Jordan , L. Torres‐Ronda , et al., “Selecting Metrics That Matter: Comparing the Use of the Countermovement Jump for Performance Profiling, Neuromuscular Fatigue Monitoring, and Injury Rehabilitation Testing,” Strength & Conditioning Journal 45 (2023): 545–553.

[22] M. J. Jordan , Z. J. McClean , P. Aagaard , K. Pasanen , H. De Brito Fontana , and W. Herzog , “Maximal Eccentric–Concentric Strength Determines Stretch‐Shortening Cycle Leg Power Across Biological Sexes,” Scientific Reports 16 (2026): 4273. 41507343 10.1038/s41598-025-34475-5 PMC12858988

[23] M. J. Jordan , P. Aagaard , and W. Herzog , “A Comparison of Lower Limb Stiffness and Mechanical Muscle Function in ACL‐Reconstructed, Elite, and Adolescent Alpine Ski Racers/Ski Cross Athletes,” Journal of Sport and Health Science 7 (2018): 416–424. 30450249 10.1016/j.jshs.2018.09.006 PMC6226549

[24] M. J. Jordan , P. Aagaard , and W. Herzog , “Lower Limb Asymmetry in Mechanical Muscle Function: A Comparison Between Ski Racers With and Without ACL Reconstruction,” Scandinavian Journal of Medicine and Science in Sports 25 (2015): e301–e309. 25212216 10.1111/sms.12314

[25] P. J. Read , S. Michael Auliffe , M. G. Wilson , and P. Graham‐Smith , “Lower Limb Kinetic Asymmetries in Professional Soccer Players With and Without Anterior Cruciate Ligament Reconstruction: Nine Months Is Not Enough Time to Restore ‘Functional’ Symmetry or Return to Performance,” American Journal of Sports Medicine 48 (2020): 1365–1373. 32293904 10.1177/0363546520912218

[26] J. J. Miles , E. King , É. C. Falvey , and K. A. J. Daniels , “Patellar and Hamstring Autografts Are Associated With Different Jump Task Loading Asymmetries After ACL Reconstruction,” Scandinavian Journal of Medicine & Science in Sports 29 (2019): 1212–1222. 31034636 10.1111/sms.13441

[27] Z. J. Mcclean , N. B. van Mossel , M. Mckenzie , et al., “Lower Extremity Asymmetry Values Derived From Multiple Strength Testing Modes Are Associated With Perceived Functional Capabilities Among University Athletes,” 2024 (2024): 5589056. 10.1155/2024/5589056 PMC11524692 39478757

[28] T. E. Moran , A. J. Ignozzi , Z. Burnett , S. Bodkin , J. M. Hart , and B. C. Werner , “Deficits in Contralateral Limb Strength Can Overestimate Limb Symmetry Index After Anterior Cruciate Ligament Reconstruction,” Arthroscopy, Sports Medicine, and Rehabilitation 4 (2022): e1713–e1719. 36312704 10.1016/j.asmr.2022.06.018 PMC9596901

[29] M. J. Jordan and C. Bishop , “Testing Limb Symmetry and Asymmetry After ACL Injury: Four Considerations to Increase Its Utility,” Strength & Conditioning Journal 46 (2023): 406–414.

[30] M. J. Jordan , P. Aagaard , and W. Herzog , “Asymmetry and Thigh Muscle Coactivity in Fatigued Anterior Cruciate Ligament—Reconstructed Elite Skiers,” Medicine and Science in Sports and Exercise 49 (2016): 11–20. 10.1249/MSS.0000000000001076 27532454

[31] T. A. Haugen , F. Breitschädel , H. Wiig , and S. Seiler , “Countermovement Jump Height in National‐Team Athletes of Various Sports: A Framework for Practitioners and Scientists,” International Journal of Sports Physiology and Performance 16 (2021): 184–189. 33217727 10.1123/ijspp.2019-0964

[32] P. Jiménez‐Reyes , P. Samozino , A. García‐Ramos , V. Cuadrado‐Peñafiel , M. Brughelli , and J. B. Morin , “Relationship Between Vertical and Horizontal Force‐Velocity‐Power Profiles in Various Sports and Levels of Practice,” PeerJ 6 (2018): e5937. 30479900 10.7717/peerj.5937 PMC6238764

[33] H. Van Eetvelde , L. D. Mendonça , C. Ley , R. Seil , and T. Tischer , “Machine Learning Methods in Sport Injury Prediction and Prevention: A Systematic Review,” Journal of Experimental Orthopaedics 8 (2021): 27. 33855647 10.1186/s40634-021-00346-x PMC8046881

[34] A. Katakam , C. Bragdon , J. H. Schwab , et al., “Development of Machine Learning Algorithms to Predict Achievement of Minimal Clinically Important Difference for the KOOS—PS Following Total Knee Arthroplasty,” Journal of Orthopaedic Research 40 (2022): 808–815. 34275163 10.1002/jor.25125

[35] K. N. Kunze , “Machine Learning Algorithms Predict Functional Improvement After Hip Arthroscopy for Femoroacetabular Impingement Syndrome in Athletes,” Journal of Bone and Joint Surgery 103 (2021): 1055–1062. 10.2106/JBJS.20.01640 33877058

[36] K. Martin , S. Wastvedt , A. Pareek , et al., “Predicting Anterior Cruciate Ligament Reconstruction Revision,” Journal of Bone and Joint Surgery 104 (2022): 145–153. 10.2106/JBJS.21.00113 34662318

[37] C. Richter , E. Petushek , H. Grindem , A. Franklyn‐Miller , R. Bahr , and T. Krosshaug , “Cross‐Validation of a Machine Learning Algorithm That Determines Anterior Cruciate Ligament Rehabilitation Status and Evaluation of Its Ability to Predict Future Injury,” Sports Biomechanics 22 (2023): 91–101. 34323653 10.1080/14763141.2021.1947358

[38] C. Richter , E. King , S. Strike , and A. Franklyn‐Miller , “Objective Classification and Scoring of Movement Deficiencies in Patients With Anterior Cruciate Ligament Reconstruction,” PLoS One 14 (2019): 1–20. 10.1371/journal.pone.0206024 PMC6650047 31335914

[39] G. S. Collins , J. B. Reitsma , D. G. Altman , and K. G. M. Moons , “Transparent Reporting of a Multivariable Prediction Model for Individual Prognosis or Diagnosis (TRIPOD): The TRIPOD Statement,” BMC Medicine 13 (2015): 1–10. 25563062 10.1186/s12916-014-0241-z PMC4284921

[40] J. B. Thorlund , L. B. Michalsik , K. Madsen , and P. Aagaard , “Acute Fatigue‐Induced Changes in Muscle Mechanical Properties and Neuromuscular Activity in Elite Handball Players Following a Handball Match,” Scandinavian Journal of Medicine & Science in Sports 18 (2008): 462–472. 18028284 10.1111/j.1600-0838.2007.00710.x

[41] R. Gathercole , B. Sporer , T. Stellingwerff , and G. Sleivert , “Alternative Countermovement‐Jump Analysis to Quantify Acute Neuromuscular Fatigue,” International Journal of Sports Physiology and Performance 10 (2015): 84–92. 24912201 10.1123/ijspp.2013-0413

[42] P. Caserotti , P. Aagaard , J. Buttrup Larsen , and L. Puggaard , “Explosive Heavy‐Resistance Training in Old and Very Old Adults: Changes in Rapid Muscle Force, Strength and Power,” Scandinavian Journal of Medicine and Science in Sports 18 (2008): 773–782. 18248533 10.1111/j.1600-0838.2007.00732.x

[43] A. Rossi , L. Pappalardo , and P. Cintia , “A Narrative Review for a Machine Learning Application in Sports: An Example Based on Injury Forecasting in Soccer,” Sports 10 (2022): 1–16. 10.3390/sports10010005 PMC8822889 35050970

[44] L. Breiman , “Random Forests,” Machine Learning 45 (2001): 5–32.

[45] H. Han , X. Guo , and H. Yu , “Variable Selection Using Mean Decrease Accuracy and Mean Decrease Gini Based on Random Forest,” in Proceedings of the IEEE International Conference on Software Engineering and Service Sciences, ICSESS (2016), 219–224.

[46] H. Berg , “Muscle Control in Elite Alpine Skiing,” Medicine and Science in Sports and Exercise 31 (1999): 1065–1067. 10416571 10.1097/00005768-199907000-00022

[47] R. Hintermeister , O. Dennis , D. Charles , C. Suplizio , G. Lange , and R. Steadman , “Muscle Activity in Slalom and Giant Slalom Skiing,” Medicine and Science in Sports and Exercise 27, no. 3 (1995): 315–322. 7752856

[48] M. Alhammoud , C. Hansen , F. Meyer , C. Hautier , and B. Morel , “On‐Field Ski Kinematic According to Leg and Discipline in Elite Alpine Skiers,” Frontiers in Sports and Active Living 2 (2020): 1–14. 10.3389/fspor.2020.00056 PMC7739787 33345047

[49] M. R. Cross , J. R. Rivière , B. Van Hooren , et al., “The Effect of Countermovement on Force Production Capacity Depends on Extension Velocity: A Study of Alpine Skiers and Sprinters,” Journal of Sports Sciences 39 (2021): 1882–1892. 33792497 10.1080/02640414.2021.1906523

[50] F. A. Faria , J. A. Dos Santos , A. Rocha , and R. D. S. Torres , “A Framework for Selection and Fusion of Pattern Classifiers in Multimedia Recognition,” Pattern Recognition Letters 39 (2014): 52–64.

[51] H. Grindem , L. Snyder‐Mackler , H. Moksnes , L. Engebretsen , and M. A. Risberg , “Simple Decision Rules Can Reduce Reinjury Risk by 84% After ACL Reconstruction: The Delaware‐Oslo ACL Cohort Study,” British Journal of Sports Medicine 50 (2016): 804–808. 27162233 10.1136/bjsports-2016-096031 PMC4912389

[52] E. King , C. Richter , K. A. J. Daniels , et al., “Can Biomechanical Testing After Anterior Cruciate Ligament Reconstruction Identify Athletes at Risk for Subsequent ACL Injury to the Contralateral Uninjured Limb?,” American Journal of Sports Medicine 49 (2021): 609–619. 33560866 10.1177/0363546520985283 PMC9938948

[53] N. F. N. Bittencourt , W. H. Meeuwisse , L. D. Mendonça , A. Nettel‐Aguirre , J. M. Ocarino , and S. T. Fonseca , “Complex Systems Approach for Sports Injuries: Moving From Risk Factor Identification to Injury Pattern Recognition—Narrative Review and New Concept,” British Journal of Sports Medicine 50 (2016): 1309–1314. 27445362 10.1136/bjsports-2015-095850

[54] C. Richter , E. King , S. Strike , and A. Franklyn‐Miller , “Objective Classification and Scoring of Movement Deficiencies in Patients With Anterior Cruciate Ligament Reconstruction (E Bergamini, Ed.),” PLoS One 14 (2019): e0206024. 31335914 10.1371/journal.pone.0206024 PMC6650047

[55] M. Taberner , N. Van Dyk , T. Allen , et al., “Physical Preparation and Return to Sport of the Football Player With a Tibia‐Fibula Fracture: Applying the Control‐Chaos Continuum,” BMJ Open Sport & Exercise Medicine 5 (2019): 1–7. 10.1136/bmjsem-2019-000639 PMC6830476 31749984

[56] R. Kotsifaki , V. Sideris , E. King , R. Bahr , and R. Whiteley , “Performance and Symmetry Measures During Vertical Jump Testing at Return to Sport After ACL Reconstruction,” British Journal of Sports Medicine 57 (2023): 1304–1310. 37263763 10.1136/bjsports-2022-106588

[57] P. J. Read , S. M. Auliffe , M. G. Wilson , and P. Graham‐smith , “Lower Limb Kinetic Asymmetries in Professional Soccer Players With and Without Anterior Cruciate Ligament Reconstruction Nine Months Is Not Enough Time to Restore ‘Functional’ Symmetry or Return to Performance,” American Journal of Sports Medicine 48 (2020): 1365–1373. 32293904 10.1177/0363546520912218

[58] C. L. Ardern , P. Glasgow , A. Schneiders , et al., “2016 Consensus Statement on Return to Sport From the First World Congress in Sports Physical Therapy, Bern,” British Journal of Sports Medicine 50 (2016): 853–864. 27226389 10.1136/bjsports-2016-096278

[59] K. E. Webster and T. E. Hewett , “What Is the Evidence for and Validity of Return‐To‐Sport Testing After Anterior Cruciate Ligament Reconstruction Surgery? A Systematic Review and Meta‐Analysis,” Sports Medicine 49 (2019): 917–929. 30905035 10.1007/s40279-019-01093-x

[60] C. Richter , N. E. O'Connor , B. Marshall , and K. Moran , “Comparison of Discrete‐Point vs. Dimensionality‐Reduction Techniques for Describing Performance‐Related Aspects of Maximal Vertical Jumping,” Journal of Biomechanics 47 (2014): 3012–3017. 25059895 10.1016/j.jbiomech.2014.07.001

[61] P. Bishop , K. Cureton , and M. Collins , “Sex Difference in Muscular Strength in Equally‐Trained Men and Women,” Ergonomics 30 (1987): 675–687. 3608972 10.1080/00140138708969760

[62] J. L. Mayhew , K. Hancock , L. Rollison , T. E. Ball , and J. C. Bowen , “Contributions of Strength and Body Composition to the Gender Difference in Anaerobic Power,” Journal of Sports Medicine and Physical Fitness 41 (2001): 33–38. 11317145

